# Survey for major viruses in commercial *Vitis vinifera* wine grapes in Ontario

**DOI:** 10.1186/s12985-018-1036-1

**Published:** 2018-08-13

**Authors:** Huogen Xiao, Mehdi Shabanian, Clayton Moore, Caihong Li, Baozhong Meng

**Affiliations:** Department of Molecular and Cellular Biology, University of Guelph, 50 Stone Road, Guelph, ON N1G 2W1 Canada

**Keywords:** Grapevine viral disease, Grapevine leafroll, Grapevine red blotch, Grapevine rugose wood, Grapevine fleck, *Vitis vinifera*, Virus survey, Ontario, RT-PCR

## Abstract

**Background:**

In recent years, the Ontario grape and wine industry has experienced outbreaks of viral diseases across the province. Little is known about the prevalence of viruses and viral diseases in Ontario. Since 2015, we have conducted large-scale surveys for major viruses in commercial wine grapes in order to obtain a comprehensive understanding of the prevalence and severity of viral diseases in Ontario.

**Methods:**

A total of 657 composite leaf samples representing 3285 vines collected from 137 vine blocks of 33 vineyards from three appellations: Niagara Peninsula, Lake Erie North Shore and Prince Edward County. These samples covered six major red cultivars and five major white grape cultivars. Using a multiplex RT-PCR format, we tested these samples for 17 viruses including those involved in all major viral diseases of the grapevine, such as five grapevine leafroll-associated viruses (GLRaV-1, 2, 3, 4, 7), grapevine red blotch virus (GRBV), grapevine Pinot gris virus (GPGV), grapevine rupestris stem sitting-associated virus (GRSPaV), grapevine virus A (GVA), grapevine virus B (GVB), grapevine fleck virus (GFkV), arabis mosaic virus (ArMV), tomato ringspot virus (ToRSV), trapevine fanleaf virus (GFLV), among others.

**Results:**

Fourteen of the 17 viruses were detected from these samples and the predominant viruses are GRSPaV, GLRaV-3, GFkV, GPGV and GRBaV with an incidence of 84.0, 47.9, 21.8, 21.6 and 18.3%, respectively. As expected, mixed infections with multiple viruses are common. 95.6% of the samples included in the survey were infected with at least one virus; 67% of the samples with 2–4 viruses and 4.7% of the samples with 5–6 viruses. The major grape cultivars all tested positive for these major viruses. The results also suggested that the use of infected planting material may have been one of the chief factors responsible for the recent outbreaks of viral diseases across the province.

**Conclusions:**

This is the first such comprehensive survey for grapevine viruses in Ontario and one of the most extensive surveys ever conducted in Canada. The recent outbreaks of viral diseases in Ontario vineyards were likely caused by GLRaV-3, GRBV and GPGV. Findings from this survey provides a baseline for the grape and wine industry in developing strategies for managing grapevine viral diseases in Ontario vineyards.

## Background

Viruses and the diseases they cause present a major roadblock to the sustainable production of quality grapes and wines [1]. Infections with individual viruses, and mixed infections with multiple viruses as is commonly seen in grapes, are responsible for considerable and perpetual economic losses to grape and wine industries worldwide [2–6]. Grapevines are known to be infected with the largest number of viruses of all cultivated plant crops. At present, over 80 distinct virus species from 17 virus families and 27 genera have been identified in grapevines [7]. The most damaging and widespread viruses are those involved in the four major disease complexes known as (1) leafroll disease complex [*Grapevine leafroll-associated virus* (GLRaV)-1, − 2, − 3, − 4, − 7 and − 13]; (2) rugose woody complex [grapevine virus A (GVA), grapevine virus B (GVB), grapevine rupestris stem pitting-associate virus (GRSPaV)]; (3) infectious degeneration and decline [grapevine fanleaf virus (GFLV), tomato ringspot virus (ToRSV), arabis mosaic virus (ArMV)]; and (4) fleck complex [grapevine fleck virus (GFkV), grapevine asteroid mosaic-associated virus (GAMaV), grapevine rupestris vein feathering virus (GRVFV), grapevine redglobe virus (GRGV)] [7]. Recently, a new disease, grapevine red blotch, caused by the DNA virus ‘grapevine red blotch virus’ (GRBV) was discovered in the USA [8, 9] and Canada [10, 11]. Another new virus, grapevine Pinot gris virus (GPGV) was identified in grapevine plants showing symptoms of chlorotic mottling and leaf deformations [12] and was soon reported worldwide from many countries in Europe, Asia, South and North America [13]. In Canada, GPGV has been reported in Ontario and British Columbia [14, 15].

The grapevine and wine industry in Canada is young, first established in the 1970s. Ontario is the largest producer of grape and wine in Canada, followed by British Columbia, Nova Scotia and Quebec [16]. The grape and wine industries constitute an important cornerstone for the economy of Ontario. Grapes rank as the second largest fruit crop in Ontario, with a farm gate value of $100 million annually. Ontario wines have garnered prestigious recognition in the international market, and produce an economic impact estimated at $4.4 billion in 2017. However, starting in 2013, the industry has experienced major outbreaks of viral diseases across the province. Infected vines showed severe symptoms suggesting infections with two disease complexes: leafroll and decline as well as the newly identified red blotch disease. Infected vineyards exhibited poor vigor, fewer and smaller berry clusters, reduced yield and sugar content, and delay and inconsistency in fruit maturity. Furthermore, viral diseases reduce winter hardiness, leading to increased susceptibility to damage or even death resulting from freezing temperatures over severe winters. Some growers had to endure total crop losses, even abandoned their vineyards. As shown in the 2016 annual report of Grape Growers of Ontario, the sales of *Vitis vinifera* wine grapes had decreased by 40% in 2014 and by 44% in 2015 as compared with that of 2013 due to the colder than normal winters in 2014 and 2015 [17]. The additional pressure of viruses and viral diseases has therefore become major concern to Ontario grape/wine community and threatens the sustainability of the grape and wine industry.

Understanding the type of viruses, their prevalence and the severity of viral diseases is the first step in winning the battle against viruses and viral diseases. However, the situation of grapevine viruses and their economic impact to grape and wine production in Canada is very limited. An earlier nation-wide survey for the distribution of four major viruses was conducted in the 1990s by Centre for Plant Health, Agriculture and Agri-Food Canada [16]. This survey targeted only four viruses, two involved in the infectious degeneration (arabis mosaic virus, ArMV and grapevine fanleaf virus, GFLV) and two involved in the grapevine leafroll,grapevine leafroll associated virus 1 (GLRaV-1) and GLRaV-3. Results of this survey showed low incidence of ArMV (0.53%) and GFLV (0.25%) but higher prevalence for GLRaV-3 (10.8%), followed by GLRaV-1 (1.67%) [16]. Though over 85% the samples included in this survey were collected from Ontario, only a small percentage of these samples (20.9%) were from *V. vinifera* cultivars. Two recent surveys were conducted in British Columbia on the distribution and genetic diversity of GRBV [11] and four viruses associated with leafroll disease [18]. GLRaV-3 was shown to be the most prevalent, being detected in 16.7% of composite samples, whereas GLRaV-1, − 2 and − 4 were detected in a small percentage of samples (< 4%). Interestingly, GRBV was shown to have a low incidence (1.6%).

Ontario is the predominant grape and wine producer in Canada. However, very little is known about the distribution and prevalence of viruses in the province. In the past two decades, there has been major shifts in the grape and wine industry in Ontario, with increased acreage of *V. vinifera* cultivars in replacement of non-vinifera grapes, such as those of the *V. labrusca* type and French-American hybrids. However, there have been no further studies on the distribution of grapevine viruses in Ontario. As a response to the disease outbreaks since 2013, a small-scale survey was conducted by McFadden-Smith and others, which revealed the presence of three grapevine leafroll-associated viruses (GLRaV-1, 2, 3), and to a lesser extent, grapevine red blotch virus in numerous vineyards (McFadden-Smith, personal communication).

To obtain a comprehensive picture on the distribution and prevalence of all major viruses in commercial vineyards across Ontario, we first developed highly effective multiplex-PCR for use in screening for a large number of viruses that are targeted in grapevine certification programs in major grape-growing countries. Here we report on the results of the survey for these viruses in *Vitis vinifera* wine grapes collected from across Ontario.

## Methods

### Sample collection and processing

#### Sampling strategies

**Fig. 1:**
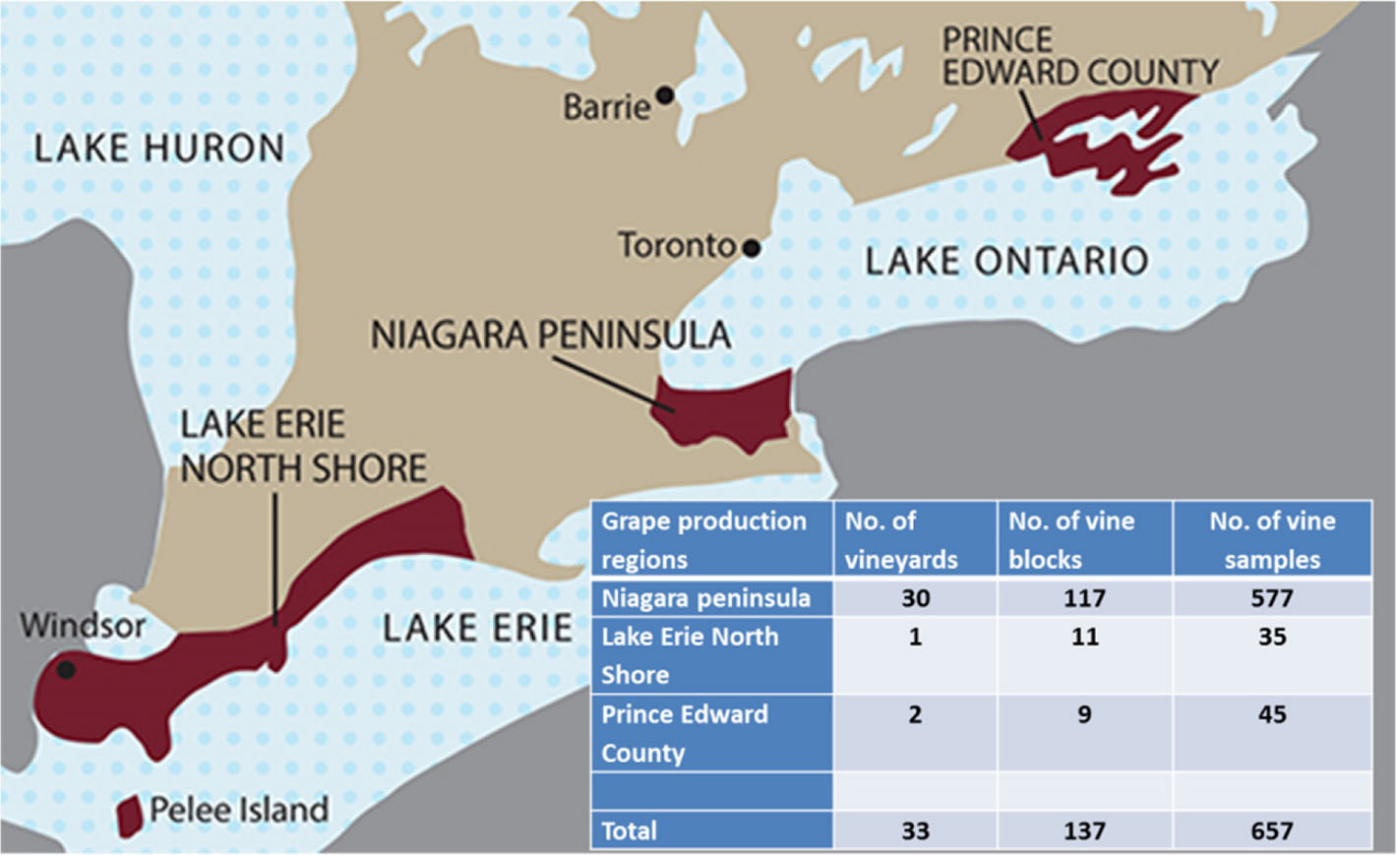
Sampling of grapevines across the three primary appellations in Ontario for use in virus survey. The map is from http://www.vintages.com/images/ontario_wine_region-map.jpg. The insert table shows the numbers of samples, numbers of vine blocks and vineyards that were sampled from the three appellations in the province. Each sample is comprised of two basal leaves per vine from five vines in a vine panel (total of 10 leaves for each composite sample)

As of 2015 the total vineyard acreage in Ontario reached 17,000 acres, of which 87%, 8% and 4% came from Niagara Peninsula, Lake Erie North Shore and Prince Edward County, respectively (Fig. 1). The five major white grape cultivars grown in 2015 were Chardonnay, Riesling, Pinot Gris, Sauvignon Blanc and Gewurztraminer and the six major red grape cultivars were Cabernet Franc, Cabernet Sauvignon, Pinot Noir, Merlot, Syrah and Gamay Noir (http://www.grapegrowersofontario.com/grape_facts). To investigate the status of virus infection in commercial wine grape vineyards in Ontario, we collected 657 composite leaf samples from a total of 3285 vines representing 137 vineyard blocks from 33 vineyards during late summer and early fall in 2015 and 2016 (Fig. 1) from the three major grape production regions: Niagara Peninsula, Lake Erie North Shore/Pelee Island and Prince Edward County (Fig. 1). The majority of the samples were collected from Niagara Peninsula as it had 87% of Ontario vineyard acreage [17] (Fig. 1). These samples covered seven red grape cultivars (Cabernet Franc, Cabernet Sauvignon, Pinot noir, Merlot, Syrah, Gamay Noir and Petit Verdot) and nine white grape cultivars (Chardonnay, Riesling, Pinot Gris, Sauvignon Blanc, Gewürztraminer, Chardonnay Musque, Chenin Blanc, Viognier and Savagnin) (Table 1). Four to ten composite samples were randomly collected from each cultivar/block according to the size of the block, and each composite sample included a total of 10 leaves, with two basal leaves from each of the five vines in a panel.

**Table 1:** Sampling of *Vitis vinifera* grape cultivars

Types of grapes	Name of cultivar	No. of vineyard blocks	No. of samples
Red grape cultivars	Cabernet Franc	23	155
	Cabernet Sauvignon	9	36
	Pinot Noir	23	92
	Merlot	9	36
	Syrah	9	51
	Gamay Noir	2	9
	Petit Verdot	1	3
Subtotal for red grapes	7	76	382
White grape cultivars	Chardonnay	16	66
	Riesling	21	117
	Pinot Gris	8	34
	Sauvignon Blanc	5	22
	Gewurztraminer	5	16
	Chardonnay Musque	3	9
	Chenin Blanc	1	3
	Savagnin	1	4
	Viognier	1	5
Subtotal for white grapes	9	61	275
Total	16	137	657

Composite sample composed of two basal leaves per vine from five vines in a panel (10 leaves in total for a composite sample)

All collected samples were ground into powder with mortar and pestle in liquid nitrogen and stored in conical tubes in a − 20 °C freezer for isolation of nucleic acids.

#### Isolation of total nucleic acids

Total nucleic acids were isolated from each of the 657 samples collected using a modified protocol we developed based on a commercial nucleic acid isolation kit [10]. The quality and concentration of the RNA preparations were assessed with a NanoDrop spectrophotometer (ND-1000, Thermo Scientific, Delaware, USA) at wavelengths of 230, 260, and 280 nm. All nucleic acid samples isolated had A260/280 and A260/230 ratio of 2.0 or above.

#### Primers for use in PCR and RT-PCR

Because most grape viruses comprise multiple strains, it is important to use broad-spectrum primers that could detect all strains of each target virus (Table 2). For this purpose, we designed these primers based on the consensus sequence of multiple genetic variants for each of these 17 target viruses. To reduce the time required for the many tests, we developed a multiplex PCR system, which would allow the simultaneous detection of multiple viruses in a single test. To ensure detection of multiple viruses in a single test, the primers were designed so that they would result in amplicons of different lengths for different viruses. Details of the primers used, the genomic region targeted, and the sizes of amplification products are listed in Table 2.

**Table 2:** Primers used for RT-PCR (or PCR) detection of target grapevine viruses

Groups	Viruses	Primers	Sequences (5′-3′)	Product (bp)	Target gene
A	GRBV	GRBaV685F	GAGGGTTGTTTGAAGATAAAG	719	CP
		GRBaV1403R	CCATAATAAACAGCGTGGTC		
	GLRaV-3	LR3-CP107F	TCTTAAARTAYGTTAAGGACGG	301	CP
		LR3-CP407R	GGCTCGTTAATAACTTTCGG		
	GLRaV-1	LR1-502F	TTGAGRGCTCTBATAAAYGAAC	379	HSP70h
		LR1-880R	CGTTMARTTCGYCKACSGACA		
	GLRaV-2	LR2-14568F	RCDATGGAGYTRATGTCYGA	525	CP
		LR2-15092R	AGCGTACATRCTYGCRAACA		
B	GVA	GVA6538F	TCTTCGGGTACATCGCCTTG	325	CP
		GVA6862R	TCRAACATAACCTGTGGYTC		
	GRSPaV	RSP35	AGRYTTAGRGTRGCTAARGC	476	Rep
		RSP36	CACATRTCATCVCCYGCAAA		
	GVB	GVB6448F	ATGGAAAATATATCCCKGATGG	603	CP
		GVB7050R	GTTAACCACCTATATYTCRACAG		
C	ToRSV	ToRSV-1	CAGGAAGGTACAGACGCC	340	CP
		ToRSV-2	ACGTCCACGTAACTTCTGG		
	GLRaV-4	LR4-13269F	GGACAATTTAGGTAATGTWGTRGCTAC	490	P23 (ORF6)
		LR4-13758R	TATCCTCAGWGAGGAARCGG		
	GFkV	GFkV5209F	GTCCTCGGCCCAGTGAAAAAG	348	Rep
		GFkV-5556R	CAGGTTGTAGTCGGTGTTGTC		
D	GFLV	GFLV3135F	TTGAGATTGGWTCYCGTTTC	558	CP
		GFLV3692R	CTGTCGCCACTAAAAGCATG		
	GAMaV	GAMaV6083F	CTCGCGCTCCTCGCATTGTT	467	CP
		GAMaV6549R	CGTGACGAGGTTGGTCCCA		
	GSyV-1	GSyV5725F	ATGATGCAACCGACCCTTCC	671	CP
		GSYV6395R	TGGAGGCTTTATTCAGAGAG		
	ArMV	ArMV2291F	CRGGTATTACGTGGGTTATGAG	292	CP
		ArMV2582R	GCTGCCTCAAACTCAGCATA		
E	GPGV	GPGV6586F	GAYATGTCGATTCGTCAGGAG	436	CP
		GPGV7021R	CGACTTCTGGTGCCTTATCAC		
	GLRaV-7	LR7-12163F	CTAGTGAATTACACCGAGAAGTC	550	CP
		LR7-12712R	GTGACTTGGCACGCATGTATC		
	GRVFV	GRVFV5646F	GTYGAARTCTCTCTCTTCTCCCA	389	CP
		GRVFV6034R	ATTATGAGAGCAACCCACTGGAAG		
	Internal reference gene	UBI-F	CCGCACTCTTGCTGATTACA	146	Ubiquitin-ribosomal protein L40–2
		UBI-R	GTGCATAACATTTGCGGCAG		

The full names of viruses that are included in this survey are provided in the list of abbreviations. *CP* capsid protein, *HSP70h* heat shock protein 70 homologue, *Rep* replicase protein

#### First-strand cDNA synthesis

A two-step RT-PCR was used throughout this virus survey. First-strand cDNA synthesis was primed with random primers using High-capacity cDNA Reverse Transcription Kit (Life technologies). The reaction mix (20 μl) includes 1000 ng of total nucleic acids, 2.0 μl RT buffer, 0.8 μl dNTP mix (100 mM), 2.0 μl RT random primers, and 1.0 μl Multiscribe™ Reverse Transcriptase (50 U/μl). The mix was incubated for 10 min at 25 °C, then for 120 min at 37 °C, followed by incubation for 5 min at 85 °C. Resulting reverse transcription reactions were used in PCR immediately or stored at − 20 °C for later use.

#### RT-PCR and PCR

The PCR reaction mix (25 μl) for single PCR contains 1 μl of cDNA (5% of the first-strand reaction, corresponding to about 50 ng of total nucleic acids), 1X PCR buffer (containing 2.0 mM MgCl_2_), 0.2 mM dNTPs, 0.2 μM each primer, and 1.0 unit of Taq DNA polymerase (GeneDireX). PCR conditions included an initial denaturation step at 94 °C for 4 min, then 40 cycles at 94 °C for 30 s, 50–55 °C (depending on the primers used) for 30 s and 72 °C for 1 min, followed by a final extension at 72 °C for 7 min. The PCR products were analyzed on 1.5% agarose gel, followed by staining with ethidium bromide.

## Results

### Fourteen viruses were detected in *Vitis vinifera* wine grapes in Ontario

We used the multiplex RT-PCR system we developed to screen for 17 viruses separated into five groups, which included five viruses that are associated with leafroll (GLRaV-1, − 2, − 3, − 4, − 7), three that are involved in rugose wood (GRSPaV, GVA, and GVB), three nepoviruses that are causal agents of the infectious degegneration and decline (ArMV, GFLV, and ToRSV), and four viruses that are associated with the fleck complex (GFkV, GAMaV, GSyV-1 and GRVFV). In addition, two newly discovered viruses, GRBV (the causal agent of red blotch) and GPGV (the putative causal agent of leaf mottle and deformation) were also included. Collectively, fourteen of them (except GLRaV-4, GLRaV-7, and GFLV) were detected (Table 3). Overall, 95.6% of the samples tested were infected with at least one virus. It is important to note that mixed infections are very common, as expected. For example, 31.9% of the samples tested positive for two viruses, 24.7% for three viruses, 10.5% for four viruses, and 4.9% for five or more viruses (Table 4). The prevalence of these 14 viruses varied greatly, ranging from 84% for GRSPaV to 0.2% for ArMV (Table 3). The most prevalent viruses are GRSPaV (84%), GLRaV-3 (47.9%), GFkV (21.8%), GPGV (21.6%) and GRBV (18.3%) (Table 3).

**Table 3:** Prevalence of major grape viruses in *V. vinifera* wine grapes in Ontario

Viruses detected	Total no. of vine samples tested	No. of positive samples	Percentage of positive samples (%)
GRSPaV	563	473	84.0
GLRaV-3	657	315	47.9
GFkV	657	143	21.8
GPGV	657	142	21.6
GRBV	657	120	18.3
GVA	657	41	6.2
GAMaV	563	34	6.0
GRVFV	563	33	5.9
GLRaV-2	657	29	4.4
GVB	657	20	3.0
GLRaV-1	657	14	2.1
GSyV-1	563	9	1.6
ToRSV	563	9	1.6
ArMV	563	1	0.2

The full names of viruses that are included in this survey are provided in the abbreviations

**Table 4:** Number and percentage of grapevine samples that are infected with a single or multiple viruses

No. of viruses in mixed infections	No. of samples with mixed infections	Percentage of samples with multiple viruses (%)
1	136	23.7
2	183	31.9
3	142	24.7
4	60	10.5
5	21	3.7
6	6	1.0
7	1	0.2

Among the five viruses associated with leafroll disease complex, three were detected in Ontario. GLRaV-3 was detected in nearly 50% of the samples, followed by GLRaV-2 (4.4%) and GLRaV-1 (2.1%). Interestingly, GLRaV-4 and the related strains, and GLRaV-7 were not detected in any of the samples we tested. We were surprised that the two newly identified viruses, GRBV and GPGV, were widely present in commercial wine grapes in Ontario. 18.3% of the samples tested positive for GRBV while an even higher percentage (21.6%) of the samples tested positive for GPGV (Table 3).

The prevalence of the three viruses associated with the RW disease complex varied depending on the virus. As expected, GRSPaV was detected in 84% of the samples. GVA and GVB, both members of the genus *Vitivirus*, were detected in 6.2% and 3.0% of the samples, respectively. Interestingly, GFLV was not detected at all while ToRSV and ArMV were present only in 1.6% and 0.2% of the samples tested, indicating very low incidence of both viruses in *V. vinifera* grapes in Ontario.

### All major *V. vinifera* wine cultivars are infected with major grapevine viruses

Though the number of viruses detected varied among the grape cultivars, overall, all major cultivars were heavily infected with multiple viruses. Cabernet Franc, Cabernet Sauvignon, Pinot Noir, Syrah, Chardonnay, Riesling, Pinot Gris and Sauvignon Blanc all tested positive for multiple viruses, only Merlot and Gewurztraminer were infected with a smaller number of viruses (Table 5). The infection rate of GLRaV-3 for Syrah, Gewurztraminer, Pinot Gris, Chardonnay, Riesling and Cabernet Franc reached nearly 50% or above and were 86.3, 68.8, 58.8, 56.1, 53 and 49.7% respectively, with only Merlot having the lowest rate of 13.9%. For the infection of GPGV Cabernet Franc, Syrah and Sauvignon had the highest infection rate of 35.5, 35.3 and 47.4% respectively, whereas Cabernet Sauvignon, Gewurztraminer and Merlot had a relatively low infection rate of 5.6, 6.3 and 8.7% respectively. For the infection of GRBV Syrah had the highest infection rate of 64.7%, followed by Cabernet Franc, Cabernet Sauvignon, Chardonnay, Pinot Gris and Sauvignon Blanc with an infection rate of 20.6–26.3%, and Riesling and Gewurztraminer with only 2 and 0% of infection rate.

**Table 5:** Prevalence of major grape viruses on major *V. vinifera* cultivars in Ontario

Viruses tested	Cabernet Franc	Cabernet Sauvignon	Pinot Noir	Merlot	Syrah	Chardonnay	Riesling	Pinot Gris	Sauvignon Blanc	Gewürz traminer
GLRaV-3	49.7	22.2	33.7	13.9	86.3	56.1	53.0	58.8	31.6	68.8
GLRaV-2	5.2	25.0	2.2	0.0	0.0	7.6	4.0	0.0	10.5	0.0
GLRaV-1	1.3	0.0	2.2	0.0	0.0	3.0	4.0	0.0	5.3	6.3
GRBV	25.8	22.2	7.6	5.6	64.7	21.2	2.0	20.6	26.3	0.0
GPGV	35.5	5.6	8.7	16.7	35.3	15.2	13.0	14.7	47.4	6.3
GVA	1.9	2.8	3.3	0.0	0.0	16.7	15.0	0.0	5.3	18.8
GVB	0.0	0.0	0.0	0.0	0.0	4.5	8.0	0.0	5.3	0.0
GRSPaV	89.8	72.2	87.0	97.2	100.0	95.5	68.0	94.1	100.0	81.3
GFkV	19.4	2.8	60.9	27.8	0.0	18.2	11.0	26.5	26.3	0.0

The full names of viruses included in this table are provided in the list of abbreviations

### Status of viral infection among vineyard blocks

As shown in Table 5, 137 vine blocks were tested for eight viruses (GLRaV-1, − 2, − 3, GRBV, GPGV, GVA, GVB and GFkV) while 127 vineyard blocks were also assayed for the remaining six viruses (GRSPaV, ToRSV, ArMV, GRVFV, GAMaV and GSyV-1). As expected, the most widely distributed virus is GRSPaV, which was detected in 92.1% of the blocks, of which 93.2% had 100% of detection in all samples collected. The second most widely distributed virus is GLRaV-3 which was detected in 68.6% of the 137 vineyard blocks tested. Importantly, over one third (38.3%) of these GLRaV-3 positive blocks had 100% infection in all the samples collected (Table 6). In sharp contrast, GLRaV-2 was detected in only 11.7% of the vineyard blocks, with a single block having all samples tested positive for the virus. Similarly, GLRaV-1 was detected in only 12 of the 137 (8.8%) blocks tested, and none of these blocks contained 100% of infections in the samples collected.

**Table 6:** Proportion of vineyard blocks surveyed that are infected with different viruses

Viruses detected	No. of vine blocks tested	No. of positive blocks	Percentage of positive blocks (%)	No. of blocks in which all sample tested positive	Percentage of blocks in which all sample tested positive^a^
GLRaV-3	137	94	68.6	36	38.3
GLRaV-2	137	16	11.7	1	6.3
GLRaV-1	137	12	8.8	0	0.0
GRBV	137	36	26.3	8	22.2
GPGV	137	62	45.3	5	8.1
GRSPaV	127	117	92.1	109	93.2
GVA	137	20	14.6	3	15.0
GVB	137	9	6.6	4	44.4
ToRSV	127	1	0.8	0	0.0
ArMV	127	1	0.8	0	0.0
GFkV	137	60	43.8	18	30.0
GRVFV	127	17	13.4	1	5.9
GAMaV	127	16	12.6	0	0.0
GSyV-1	127	7	5.5	0	0.0

The full names of viruses that are included in this survey are provided in the abbreviations

^a^The percentage is obtained by the number of blocks, in which all the samples are positive for a given virus, divided by the number of positive blocks for the virus

The third most widely distributed virus is GPGV. This virus was detected in 62 (45.3%) of the 137 vineyard blocks tested. Interestingly, only five of these 62 positive vineyard blocks contained samples with the infection rate of 100% (Table 6). Immediately following GPGV is GFkV, which was detected in 60 (43.8%) of the 137 blocks, with 30% of the positive blocks containing samples of 100% of infection. GRBV, on the other hand, was detected in 36 (26.3%) of the 137 vineyard blocks. Interestingly, 8 of these 36 positive blocks exhibited 100% of infection among the samples that were tested.

GVA was detected in 20 (14.6%) of the 137 blocks, three of which had 100% infections among all the samples collected. GVB was detected at a much lower frequency, with nine (6.6%) of the 137 vineyard blocks being tested positive for this virus. Interestingly, four of these nine blocks contained 100% of infection among the samples collected (Table 6). The two nepoviruses, ToRSV and ArMV, were detected in only one block each (Table 6), suggesting an isolated introduction of both viruses, likely by way of planting materials that were used at time of vineyard establishment.

### Virus status in vineyard blocks of different ages

Of the 657 samples, 476 samples were collected from vineyard blocks for which the year of plantation was known. To probe into possible correlation of virus infection status and time of vineyard establishment, these 476 samples were divided into three categories: those planted in the period between 1974 to 1990, those that were established in the period between 1991 to 2005, and those that were established since 2006 (Table 7). Several distinctive trends can be observed. GRSPaV was detected in > 90% of the samples regardless of the age of the vineyard blocks. This high level of prevalence is in line with its ubiquitous distribution among commercial grapevines (for a review, see reference [27]) and demonstrated extensive presence of this virus among planting materials. A very different trend was discerned for GLRaV-3. This virus was detected in a vast majority (71.1%) of samples from vineyard blocks that were established between 1974 and 1990. In contrast, its prevalence declined in younger vineyard blocks. For instance, 55.7% of the samples from vineyard blocks planted between 1991 and 2005 were positive for the virus, while only 37.8% of the samples from vineyard blocks planted since 2006 tested positive for GLRaV-3 (Table 7). The opposite trend was seen for GPGV. This virus was detected among only 13.3% of the samples from vine blocks established between 1974 and 1990. On the other hand, GPGV had a prevalence of 21.8% in samples from vines planted between 1991 and 2005 and 25.7% in samples from vines planted since 2006 (Table 7). Both GVA and GVB were more widely distributed in samples from older vine blocks established before 1990 with very low incidence in samples from younger vineyards (Table 7).

**Table 7:** Prevalence of major grape viruses in *V. vinifera* grapes based on period of vineyard establishment

Year of Planting	No. of samples tested	GRSPaV	GLRaV-3	GPGV	GRBV	GVA	GVB	GLRaV-2	GLRaV-1
1974–1990	45	91.1	71.1	13.3	2.2	53.3	22.2	6.7	8.9
1991–2005	357	92.7	55.7	21.8	17.9	3.6	2.8	2.0	2.0
2006–2016	74	95.9	37.8	25.7	6.8	1.4	0.0	0.0	0.0
Total	476	443	259	103	70	44	20	10	11

The full names of viruses that are included in this survey are provided in the list of abbreviations

Shown are the percentages of samples tested positive for each virus over the total number of samples collected from grapevines planted in a specific period of years

The trend of distribution of GRBV in vines of different age groups is puzzling. This virus was present in only 2.2% of the samples from vines planted before 1990, indicating low rate of infection in the earlier planting materials. However, 17.9% of the 357 samples from vines planted in the period between 1991 and 2005 tested positive for the virus. Surprisingly, only 6.8% of the samples from vines planted since 2006 were infected by this virus (Table 7).

## Discussion

This work represents the most comprehensive and up to date survey for all major viruses in *V. vinifera* wine grapes in Ontario, the major grape and wine producer in Canada. In recent years, there have been outbreaks of viral diseases in commercial vineyards in Ontario, resulting in severe damage and even total crop losses. To investigate the viruses that may be involved in the disease outbreaks, a province-wide survey for commonly targeted viral pathogens in commercial *V. vinifera* wine grape vineyards in Ontario were carried out in 2015 and 2016. Using a multiplex RT-PCR format we recently developed, we have tested for 17 viruses from 657 composite samples representing 3285 vines collected from the three major grape-growing regions in the province. All but three of these viruses were detected. These viruses have varying degrees of prevalence, ranging from 0.2 to 84% (Table 3). Importantly, the most destructive viruses involved in leafroll, red blotch, and rugose wood were all widely detected. Mixed infections are very common. The most widely distributed viruses include GLRaV-3 (47.9%), GRBV (18.3%), GPGV (21.6%), GFkV (21.8%) and GRSPaV (84%), which are present in large proportions of vineyard blocks we have surveyed. We also showed that all major wine cultivars are heavily infected with multiple viruses.

It is important to note that much higher prevalence of both GLRaV-3 and GRBV was detected in Ontario compared to British Columbia. For instance, only 1.6% of the 2000 samples tested positive for GRBV in British Columbia [11] while GLRaV-3 was detected in < 17% of *V. vinifera* wine grape samples [18]. Possible reasons for these discrepancies may include differences in the infection status of propagating materials used to establish vineyards, difference in the duration and efficiency of transmission by insect vectors, and efficiencies of nucleic acid isolation systems and test methods. For example, we replied entirely on PCR-based tests for both viruses whereas ELISA was used for the survey of GLRaV-3 in British Columbia.

What factors were responsible for the heavy infection of commercial wine grapes with multiple viruses in Ontario vineyards? Undoubtedly, one of the main factors would be the use of uncertified, virus-infected planting material as illustrated by the high percentage of blocks with 100% infection rate in this survey (Table 6). Another potentially important factor would be the presence of arthropod vectors in Ontario, which have been confirmed in other countries to transmit some of these viruses. It has been reported that GLRaV-3 can be transmitted by multiple species of mealybugs (family *Pseudococcidae*) and soft scales (family *Coccidae*) [19–24]. GRBV was reportedly transmitted under greenhouse conditions by the three-cornered alfalfa treehopper, *Spissistilus festinus* (Say) [25]. However, it remains unknown if it is the only vector or one of the vectors that transmit GRBV in nature. In a recent study, Poojari et al. [18] demonstrated the presence of grape mealybug (*Pseudococcus maritimus*), the soft scale insect *Parthenolecanium corni* and likely other species in British Columbia. The grape mealybug and cottony maple scale (*Neopulvinaria innumerabilis*) were also detected in Ontario (McFadden-Smith, personal communication). The higher incidence of GLRaV-3 in older vineyards compared to younger vineyards points to the possibility of GLRaV-3 spread by vectors in Ontario, resulting in higher infection rate over time. Interestingly, the prevalence of GLRaV-3 in Ontario as surveyed in the mid 1990s was 3.42% among samples collected from *V. vinifera* grape cultivars [16]. A similar trend is also observed for GVA and GVB. Interestingly, both GVA and GVB are known also to be transmitted by mealybugs. Much further work is required in order to identify the vectors that can transmit these important viruses and the role they play in the spread of these viruses and their diseases in Ontario.

The disease Grapevine leaf mottling and deformation (GLMD) was first reported on Pinot Gris and Pinot Blanc in Northern Italy in 2003. Symptoms of GLMD include short internodes, mottling and deformation of leaf blades, smaller clusters and reduced yield [13]. In 2012, the genome of its putative causal agent, GPGV, was determined through next generation sequencing [12]. Within a short period of time following its discovery, the distribution of GPGV has reached a global scale as it has been reported from 16 countries so far [13], including Canada [14, 15]. Based on the data from this survey, GPGV was one of the most prevalent viruses, which was detected in different *V. vinifera* wine grapes grown in Ontario, with a prevalence of 21.6% (Table 3). It was recently reported that, in controlled conditions, GPGV was transmissible by the eriophyid mite *Colomerus vitis* in Italy [26]. It remains unknown if similar vectors exist in Ontario or other viticulture regions of Canada.

Our survey results suggest that GRBV and GPGV were more recently introduced into Ontario. This is based on the observation that in vineyards that were established between 1974 and 1990, both viruses were present in a small percentage of samples. In contrast, both viruses were detected in much higher percentage of grapes that were planted since 1991. For example, only 2.2% of grapes planted during 1974–1990 tested positive for GRBV. In sharp contrast, GRBV was detected in 17.9% of samples from vineyards established during 1991–2005. Similarly, the incidence of GPGV increased from 13.3% in grapes planted between 1974 and 1990 to 21.8% in grapes planted in the period of 1991–2005, to 25.7% in vines planted since 2006. These results may suggest increased introduction of both viruses along with infected planting materials in the past two decades. Both GRBV and GPGV were new viruses that were recently identified [8, 9, 12]. It is most likely that both viruses might have already existed in other grape growing regions for quite some time prior to their identification. As these two viruses were not included in the early list of viruses that were regulated or targeted in the clean stock and certification programs, the propagation material used may have been infected with either virus and became a source of further infection in newly established vineyards. The high percentage of vineyard blocks in which 100% of the composite samples were positive for GRBV (Table 5) lends support to this possibility. It is therefore of critical importance for the nurseries to survey their propagation material to ensure freedom of GRBV and GPGV, as well as other major viruses.

GRSPaV is the most widespread virus with a worldwide distribution [27]. It is not surprising that GRSPaV was detected in 84% of the samples tested. Though GRSPaV has been associated with RSP [28–30] and vein necrosis [31], definitive evidence that it is the causal agent of these diseases is still lacking. It was reported that GRSPaV infection has no major impact on growth and yields [32] and that it may be even beneficial to the grapevine host by enhancing tolerance against drought [33, 34]. It is important to note that GRSPaV comprises a family of strains that differ vastly in genome sequence and possibly also in pathogenicity. In line with this, strains SY and PN were detected in declining vines of cultivars Syrah and Pinot Noir vines [35–37]. Furthermore, GRSPaV infection was correlated to decreased defense responses (Gambino et al. [33]), which may render the vine infected with GRSPaV more susceptible to infection by other viruses. As revealed in this survey, a vast majority of the samples were infected GRSPaV in combination with one of more other viruses. The real economic impact of GRSPaV, either alone or in combination with other viruses with multiple viruses, remains to be determined.

Several viruses of the family *Tymoviridae* that are associated with the Fleck complex [38] were also detected in this survey. GFkV was detected in 21.8% of the samples with the other three viruses being detected much more rarely. GFkV was also commonly detected in British Columbia, with 29.7% of the samples tested positive for this virus [39]. The real economic impact of GFkV, and the related viruses, awaits to be seen.

Several important questions remain to be addressed. Non-vinifera grapes (table grapes, juice grapes, and hybrid wine grapes) constitute an important part of Ontario’s grape/wine industry. These locally important, non-vinifera grapes had been widely planted in the province, though large acreages of these grapes have been replaced by vinifera grapes in recent decades. As a result, both types of grapes are often grown in close vicinity to each other. As such, they could serve as reservoirs of viruses for infecting commercial wine grape vineyards through vector transmission. While this survey as well as the two surveys conducted in British Columbia [11, 18] provided compelling evidence for the wide distribution of major viruses in vinifera wine grapes, virtually no information is available pertaining to the distribution of viruses among non-vinifera grapes. We are currently conducting surveys for major viruses in these non-vinifera grapes in order to obtain a holistic assessment on the scope and magnitude of viral diseases in Ontario. This is important as all types of grapes, regardless of their genetic background or uses, would serve as host to many of the viruses that are destructive to the grape and wine industry, and consequently, would serve as natural reservoirs for the spread of viruses.

The ultimate solution to viruses and viral diseases is the generation and broad implementation of propagating materials that are free of all major viruses. This has been practiced in many grape-growing regions in the world, including the European Union, California and other states of the USA, and Australia. These survey results demonstrate the wide distribution of major viruses and the severity of viral diseases, urging the establishment of a nationwide clean stock certification program in Canada. In the meantime, it would be prudent to screen planting materials for certain most pathogenic viruses, producing the so called “best available materials’ for use in the establishment of new vineyards or replantation in existing vineyards before the full implementation of clean stock certification program. It is also crucial to identify biological vectors present in Ontario that are responsible for the transmission of highly detrimental viruses, which would allow the establishment and implementation of integrative strategies for the effective control of viral vectors.

## Conclusions

Here, we report on the results of a large-scale survey for 17 viruses in *V. vinifera* wine grapes in Ontario. Fourteen viruses were detected including viruses involved in leafroll, red blotch, among others. This is the first and most comprehensive survey ever conducted in Ontario. The predominant viruses are GLRaV-3, GRBaV, GPGV, GRSPaV, and GFkV with lower prevalence for the other viruses. The presence of viral diseases likely resulted from the use of infected planting material, combined with possible local spread by insect vectors, and the lack of rouging infected vines from vineyards. Our survey results stress the urgent need for the development and implementation of clean stock certification programs in Canada. We also emphasize the importance of public outreach activities to educate all stakeholders on the recognition of viral diseases and effective management strategies. The information from this work provides science-based guidelines for the grape and wine industries, federal and provincial government agencies, diagnostic facilities, and research institutions in devising and implementing effective measures for combatting these viral diseases in commercial vineyards in order to mitigate the economic losses due to viral diseases.

## Abbreviations

ArMV: Arabis mosaic virus
GAMaV: grapevine asteroid mosaic-associated virus
GFkV: grapevine fleck virus
GFLV: grapevine fanleaf virus
GLRaV-1, 2, 3, 4, 7: grapevine leafroll-associated virus 1, 2, 3, 4, 7
GPGV: grapevine Pinot gris virus
GRBV: grapevine red blotch virus
GRSPaV: grapevine rupestris stem sitting-associated virus
GRVFV: grapevine rupestris vein feathering virus
GSyV-1: grapevine Syrah virus − 1
GVA: grapevine virus A
GVB: grapevine virus B
ToRSV: tomato ringspot virus
